# Surveillance for Intracellular Antibody by Cytosolic Fc Receptor TRIM21

**DOI:** 10.3390/antib5040021

**Published:** 2016-11-02

**Authors:** William A. McEwan

**Affiliations:** MRC Laboratory of Molecular Biology, Francis Crick Avenue, Cambridge CB2 0QH, UK; wmcewan@mrc-lmb.cam.ac.uk

**Keywords:** intracellular antibody, TRIM21, adenovirus, virus neutralization, auto-immunity

## Abstract

TRIM21 has emerged as an atypical Fc receptor that is broadly conserved and widely expressed in the cytoplasm of mammalian cells. Viruses that traffic surface-bound antibodies into the cell during infection recruit TRIM21 via a high affinity interaction between Fc and TRIM21 PRYSPRY domain. Following binding of intracellular antibody, TRIM21 acts as both antiviral effector and sensor for innate immune signalling. These activities serve to reduce viral replication by orders of magnitude in vitro and contribute to host survival during in vivo infection. Neutralization occurs rapidly after detection and requires the activity of the ubiquitin-proteasome system. The microbial targets of this arm of intracellular immunity are still being identified: TRIM21 activity has been reported following infection by several non-enveloped viruses and intracellular bacteria. These findings extend the sphere of influence of antibodies to the intracellular domain and have broad implications for immunity. TRIM21 has been implicated in the chronic auto-immune condition systemic lupus erythematosus and is itself an auto-antigen in Sjögren’s syndrome. This review summarises our current understanding of TRIM21’s role as a cytosolic Fc receptor and briefly discusses pathological circumstances where intracellular antibodies have been described, or are hypothesized to occur, and may benefit from further investigations of the role of TRIM21.

## 1. Introduction

Antibodies are secreted by plasma cells into the extracellular environment whereupon they are excluded from the cytoplasmic environment by cell-limiting and endosomal membranes. IgG molecules that become internalized via endocytosis may be degraded in the endo-lysosomal pathway or returned to circulation by FcRn after entering the recycling endosome pathway [1]. In this way, IgG molecules are maintained at high concentration in plasma with a half-life of around 3 weeks. The physiological exclusion of antibody from the cytosolic compartment allows its mislocalization to the cytoplasm to serve as a proxy for danger. The receptor for antibody in the cell interior is tripartite motif 21 (TRIM21) which is widely expressed in the cytoplasm. TRIM21 was first demonstrated to interact with IgG1 Fc domain in a yeast two-hybrid screen [2] but the significance of this interaction remained cryptic until adenovirus particles were demonstrated to import antibodies during infection [3]. Upon entry to the cytoplasm, antibody-coated virus particles are rapidly detected owing to the ultra-high affinity interaction between TRIM21 and antibody Fc [4,5]. The response to intracellular antibody is manifest as two simultaneous events: the rapid destruction of antibody-bound particles and the activation of innate immune signalling pathways [3,6]. A role for TRIM21 is emerging in which it surveys the intracellular environment for antibody, which serves as a potent proxy signal for compromised cellular integrity when in the cytoplasm. TRIM21 represents a non-canonical immunoreceptor by virtue of its atypical cytoplasmic location, near-universal tissue expression, and dual effector–sensor functionality.

## 2. TRIM21 Is an Ultra-High Affinity Fc Receptor

**Figure 1:**
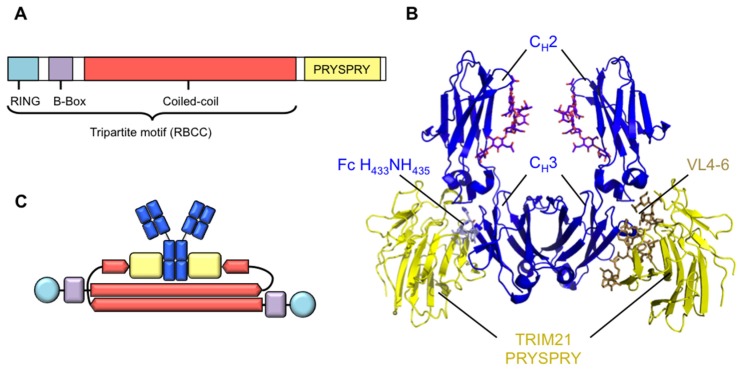
Molecular architecture of TRIM21. (**A**) TRIM21 contains a tripartite or RBCC motif, bearing an N-terminal RING domain, a B-Box and a coiled-coil domain, which consists of heptad and hendecad repeats that form an extended helical structure. TRIM21 bears an additional C-terminal PRYSPRY domain responsible for binding IgG Fc; (**B**) Crystal structure of IgG Fc (**blue**) complexed to TRIM21 PRYSPRY domain (**yellow**). The H433N434H435 motif in CH3 is accommodated in the PRYSPRY binding pocket created by variable loops (VL) 4, 5 and 6. PDB: 2IWG; (**C**) Predicted domain organisation of TRIM21 dimers. N-terminal RING and B-Box domains are held at opposing ends of the assembly due to anti-parallel association of the coiled-coil domains. PRYSPRY domains are predicted to be held centrally. Colour scheme as in (**A**).

TRIM21 belongs to the large tripartite motif (TRIM) family of proteins, which number around 100 in the human genome [7,8]. The family shares a common domain organization comprising an N-terminal RING domain with E3 ubiquitin ligase activity, at least one B-box and a helical coiled-coil domain, together forming the tripartite or RBCC (RING-Box-Coiled Coil) motif (Figure 1A). Many TRIM proteins are defined by an additional C-terminal domain, often capable of heterotypic protein–protein interactions. In the case of TRIM21, along with about half of the TRIM family, the C-terminal domain is a PRYSPRY domain. The PRYSPRY itself is a fusion between two more ancient domains, the N-terminal PRY element and larger, C-terminal B30.2/SPRY domain [9]. The B30.2/SPRY is a beta-sandwich comprising two layers of anti-parallel beta strands. Variable loops connect these beta-strands and form a deep binding pocket (Figure 1B). The antibody binding site for TRIM21 is in the CH2 and CH3 domains of Fc, centring on residues H_433_NH_435_ in the CH3 domain which are accommodated into the PRYSPRY pocket during binding. The TRIM21 binding site therefore overlaps with those of FcRn and the superantigen *Staphylococcus aureus* protein A, but is distinct from the FcγRI site. Recent crystallographic, biochemical and SAXS studies suggest that the coiled-coil domains, responsible for forming dimers of TRIM molecules, are held in an antiparallel arrangement, i.e., the N-termini of the two molecules are at opposite ends of the dimer (Figure 1C). The two PRYSPRY domains are predicted to be held in proximity at the centre of the molecule [10,11,12]. Dimers of TRIM21 bind antibody with equimolar stoichiometry such that one TRIM21 dimer engages one antibody molecule [4]. The dissociation constant of this interaction is approximately 600 pM making it, alongside the IgεR–IgE interaction, the highest affinity antibody–receptor interaction in humans and the highest for IgG [3]. In addition to its interaction with IgG, TRIM21 can bind to the IgM and IgA antibodies [3,13]. TRIM21 is unique in this respect, as other receptors are limited to a single class of antibody. The interaction between TRIM21 and IgM or IgA is of lower affinity than the interaction with IgG but may represent important protection in early adaptive immune responses, or at mucosal surfaces respectively.

Homologues of an ancestral TRIM gene, *TRIM37*, are found in diverse eukaryotic taxa including plants and fungi, making the TRIM family evolutionarily ancient [14]. However, TRIM proteins that bear a C-terminal PRYSPRY domain are unique to vertebrates. The PRYSPRY-bearing group of TRIMs underwent expansion via gene duplication events early in mammalian evolution, giving rise to a conserved syntenic region bearing related TRIM genes on human chromosome 11 that includes *TRIM21*, *TRIM6*, *TRIM22* and *TRIM5*, whose alpha isoform encodes an antiretroviral factor. During a similar era in mammalian evolution, IgG and IgE are proposed to have arisen from a duplication of the ancestral IgY [15]. Thus, whilst proteins that fulfil a similar role in non-mammalian vertebrates may exist, the TRIM21–IgG interaction is unique to, and likely universal in, mammals.

## 3. Correlates of Immunity to Viral Infection

Over the past century, antibodies have been shown to elicit a wide variety of extracellular functions including complement fixation, antibody-dependent cellular cytotoxicity (ADCC), clearance of pathogens through stimulation of phagocytosis and blocking the entry of pathogens to target cells. Determining how antibodies protect against a given pathogen is complex and idiotypic adaptive responses mean that the mechanism of protection against the same pathogen may vary between individuals. However, for most viral infections, the best correlate of immunity is a neutralizing antibody response [16,17]. In this case, neutralization is defined as the in vitro reduction in viral titre following virus binding to antibody, thus excluding mechanisms where antibody does not bind directly to virus particles (such as ADCC) and is normally assayed in the absence of active complement [18]. A neutralizing antibody response has therefore become one of the primary aims of vaccination strategies and is routinely used as a surrogate marker of host immunity.

**Figure 2:**
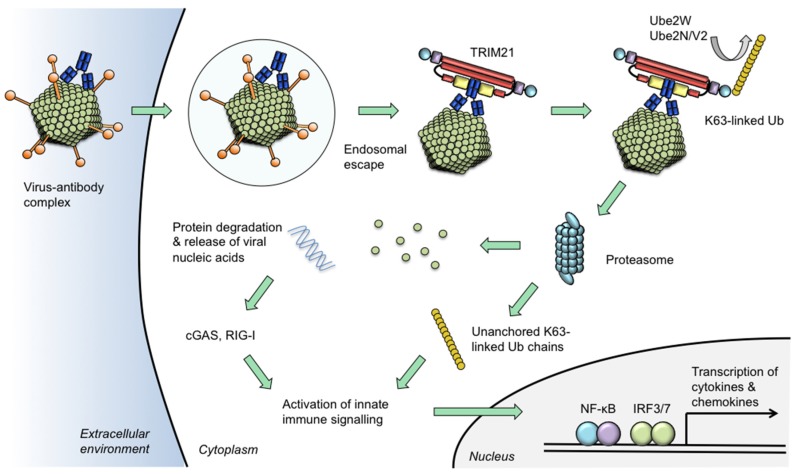
Model of TRIM21 neutralization and signalling activities. Certain viruses traffic antibodies attached to their capsids into the cytoplasm following escape from the endosome. Intracellular antibodies are bound by TRIM21 at 1:1 stoichiometric ratio. TRIM21 catalyses its auto-ubiquitination via mono-ubiquitination by Ube2W and K63-linked chain extension by Ube2n/Ube2V2. The proteasome degrades viral components causing the neutralization of infection. Concurrent with degradation, unanchored ubiquitin chains are released via the activity of proteasome-resident de-ubiquitinating enzyme POH1. Activation of NF-κB and IRF3/7 ensues which is attributable to both a direct effect of TRIM21 and through the release of viral genomes which are detected by nucleic acid sensors cGAS (for DNA) or RIG-I (for RNA). Activation of innate immune signalling stimulates production of pro-inflammatory chemokines including CCL5 and CXCL10 and cytokines such as IL-6 and interferon-β. In this way, TRIM21 uses intracellular antibody to prevent infection via its neutralization effector mechanism and to induce a transcriptional state that is refractory to viral replication in neighbouring cells.

Experiments with the non-enveloped DNA virus, adenovirus type 5, demonstrated that neutralization was reduced to minimal levels when TRIM21 was depleted by siRNA or genetic knockout [3,19]. This effect was observed for pooled human serum IgG (where neutralizing titre is generally high due to the widespread seroprevalence of adenovirus type 5 [20]) as well as monoclonal and polyclonal antibody preparations. These finding were supported by in vivo experiments, where infection with mouse adenovirus type 1 (MAV-1) demonstrated that TRIM21 expression is required for full levels of protection conferred by passively transferred immune sera [21]. These findings have established that virus neutralization operates via two broad mechanisms: (1) preventing entry of virus or viral components to the cell; and (2) in the cytosolic environment after engaging TRIM21 [18] (Figure 2). Furthermore, the findings demonstrate that, far from being passive players in the process of antibody neutralization, the cell and its molecular machinery are able to mount a last-line of defence that contributes to the survival of mammalian hosts during virus infection.

## 4. A Model System for Intracellular Neutralization

Much of the work to date on the mechanism of TRIM21 neutralization and signalling activity has relied on adenovirus, a non-enveloped DNA virus with a capsid diameter of ~90 nm. Adenovirus particles are endocytosed following interactions between virus fibre and attachment receptors, such as the coxsackie and adenovirus receptor (CAR), and between penton base and entry receptors, αβ-integrins, reviewed in [22]. This process requires membrane wounding by adenovirus protein VI, which stimulates a cell-mediated repair and uptake response [23]. Virus particles escape leaky endosomes and are released into the cytosol with an intact capsid structure [24]. As long ago as 1985 it was shown that hexon antisera neutralized adenovirus infection without preventing endocytosis of virus particles [25,26]. The hexon-specific monoclonal antibody 9C12 was similarly found to inhibit virus infection after entry but before nuclear penetration [27,28]. Following the discovery of neutralization by TRIM21, the dependencies of 9C12 neutralization were reinvestigated and found to be almost entirely reliant on TRIM21, establishing a robust model for intracellular neutralization [19]. The dependence of 9C12’s activity on a direct interaction with TRIM21 was further confirmed, as mutations in the Fc H_433_NH_435_ of 9C12 heavy chain reduced neutralization. The neutralization of adenovirus type 5 by 9C12 therefore represents a valuable tool that has allowed the detailed study of TRIM21-dependent intracellular neutralization [13,19,29,30,31,32]. 9C12 has recently been humanized, permitting analysis of neutralization in the context of the cognate human Fc:human PRYSPRY interaction [30]. A panel of point mutations in the humanized 9C12 Fc domain were generated that reduce the affinity of the interaction with TRIM21. From analysis of these antibodies, it was concluded that the signaling response of TRIM21 requires a high affinity Fc:PRYSPRY interaction but that the neutralization response is less sensitive to reduced affinity. This finding implies that pro-inflammatory signaling is more tightly regulated than neutralization and has implications for IgM and IgA, which bind with lower affinity to TRIM21 than IgG, as they may preferentially evoke neutralization over a signaling response.

## 5. TRIM21 Exerts Potent Neutralization of Virus Infection

The number of antibodies required to neutralize a virus particle has been a source of contention since some of the earliest neutralization experiments. Attempts to quantify directly the number of antibodies required for neutralization have found that low numbers of antibodies can be sufficient: 5–6 antibodies per virus particle are sufficient to neutralize rhinovirus [33]; 1–4 for poliovirus [34,35]. One study reported that 1.4 antibodies were sufficient to neutralize adenovirus in Hela cells [36], though it has since been questioned why infectivity was completely inhibited in this study when Poisson analysis implies that 25% of virions should remain unbound by antibody [18]. Using the model system of adenovirus neutralization by 9C12, it was demonstrated that ~10 antibodies per virus are required for neutralization in a HeLa cell [19]. Manipulation of TRIM21 levels be siRNA or IFN treatment changed the number of antibodies required for neutralization. Under conditions of IFN treatment in mouse cells, neutralization by 9C12 approached its theoretical maximum, with 1.6 antibodies being sufficient. Importantly, manipulating the levels of TRIM21 resulted in a series of straight-line neutralization curves with varying slope. These data support a model in which neutralization proceeds incrementally, i.e., that each antibody that binds to virus contributes equally to neutralization, rather than a threshold number being required (reviewed in [18,37]). The magnitude of the contribution of each antibody is in turn determined by the intracellular environment, most prominently the levels of TRIM21. The discovery of TRIM21 neutralization therefore has the potential to provide a mechanistic underpinning to previous observations of low stoichiometric neutralization and provides impetus for examining the dependencies of neutralization for numerous viruses.

## 6. Intracellular Antibody Is a Danger Signal

Eukaryotic cells possess numerous sensors for detecting the presence of infection [38]. Detection of intracellular pathogens can stimulate autocrine signalling pathways to induce a state that is refractory to pathogen replication. It can also alert neighbouring cells and the professional immune system by stimulating the production of cytokines and chemokines. Detection strategies in the intracellular domain typically rely on the sensing of pathogen-associated molecular patterns (PAMPs). However, TRIM21 activates the NF-κB, AP-1 and IRF signalling pathways independent of PAMPs by sensing antibody that is translocated into the cell during adenovirus infection [6] (Figure 2). The independence of PAMPs was demonstrated by showing that non-pathogenic substrates such as antibody-coated latex beads are fully capable of eliciting TRIM21-dependent signaling [6]. Sensing by TRIM21 is observed in cells that do not belong to the professional immune system, including primary human lung fibroblasts, mouse embryonic fibroblasts and commonly used human cell lines HeLa and HEK293. Activation is also observed in professional immune cells, such as macrophages, but is accompanied by responses from other Fc receptors. Sensing by TRIM21 therefore allows the recognition of intracellular pathogens without relying on PAMPs. Importantly, by using antibody/TRIM21 as a proxy marker for viral infection, the cell can detect infection at much lower multiplicity than virus in the absence of antibody [6,31]. TRIM21 signalling elicits the production of cytokines and chemokines including IFN-β1, IL-6, TNF, CCL5 and CXCL10 [6]. This response is also seen during infection of mice with antibody-bound adenovirus particles, where an inflammatory cytokine response is observed 6 hours post-infection that is impaired in the *Trim21*^−/−^ genetic background [31]. Media transfer experiments demonstrate that the soluble inflammatory stimuli released following TRIM21 signalling are sufficient to induce an antiviral state in naive cells [6]. Other pathogens aside from adenovirus have been demonstrated to induce TRIM21 signalling through the translocation of antibodies to the intracellular domain including non-enveloped viruses human rhinovirus 14 and feline calicivirus and the facultatively intracellular bacteria *Salmonella enterica* Typhimurium [6,31]. The detection of serum proteins in the intracellular domain appears to be a widely employed strategy as an alternative to direct sensing of PAMPs: complement component C3, which becomes covalently bound to pathogens in serum, activates a similar response in the intracellular environment [39]. By recognising the translocation of serum proteins, the cell can make use of the rapidly evolving and broadly acting humoral immune response to identify pathogens as an alternative to relatively invariant germline-encoded receptors.

## 7. Molecular Basis of TRIM21 Activity

TRIM21 neutralization culminates in the destruction of the virus-antibody complex at the proteasome [3]. Proteasome inhibitors prevent this degradation activity and prevent neutralization. The RING domain of TRIM21 possesses E3 ubiquitin ligase activity, which is required for both its signalling and neutralization functions [6,29]. During in vitro ubiquitination reactions, the E2 ubiquitin-conjugating enzyme Ube2W catalyses the mono-ubiquitination of TRIM21. Subsequently, ubiquitinated TRIM21 becomes a substrate for the Ube2N/UbeV2 E2 heterodimer, which catalyses ubiquitin chain extension [29]. A requirement for both Ube2W and Ube2N was demonstrated in human cell lines for both signalling in response to adenovirus:antibody and neutralization of adenovirus by 9C12. The model of TRIM21 mono-ubiquitination preceding chain extension was supported by experiments in which depletion of Ube2W prevented poly-ubiquitination of TRIM21. How the enzymatic activity of TRIM21 is able to elicit both signalling and neutralization has not been fully resolved. However, the proteasome-resident deubiquitinating enzyme POH1 (Rpn11 in yeast) was shown to be essential for both neutralization and signalling activities of TRIM21 [29]. POH1 cleaves ubiquitin chains *en bloc* by hydrolysing the substrate-proximal isopeptide bond, releasing substrates for translocation into the proteolytic chamber of the proteasome [40,41]. This activity is hypothesized to permit proteasomal destruction of the antibody:virus complex whilst simultaneously liberating free ubiquitin chains that act as a pro-inflammatory messenger [42,43] (Figure 2).

The adenovirus-antibody complex is a compact, proteinaceous megadalton particle with a diameter greater than 90 nM, whereas the proteasomal pore is approximately 2 nM in diameter. The virus-antibody complex therefore represents a challenging substrate for degradation by the ubiquitin-proteasome system (UPS). p97 or valosin-containing protein (VCP) is a ubiquitously-expressed homo-hexameric AAA ATPase that can exert unfoldase and segregase activity upstream of proteasomal degradation [44]. Inhibition or depletion of VCP prevents neutralization in a similar manner to proteasome inhibitors [32]. These results suggest that VCP may aid in the remodelling of the virus-antibody complex prior to degradation by the proteasome. Interestingly, this VCP requirement is substrate-specific, since degradation of IgG heavy chain expressed in the cytoplasm is prevented by inhibition of the proteasome but not of VCP. Thus the challenging nature of the virus-antibody complex as a proteasomal substrate may be the trigger for VCP recruitment, a phenomenon that has been observed in other settings [45]. The stimulus for activation of TRIM21 ubiquitin ligase activity is presumed to result from its binding to antibody, possibly in the form of a conformational change or higher-order assembly of TRIM21 induced by binding to antibodies. The catalytic state of RING domains from TRIM proteins is governed by their oligomeric state [46], making such a model plausible. However, the precise mechanism that governs TRIM21 activation remains elusive.

## 8. TRIM21 Neutralization Potentiates Nucleic Acid Sensing

Viruses use their capsid structures to physically protect their genomes and to hide them from host immune sensors. Nucleic acid sensors such as TLR-3 and TLR-9 recognize double stranded RNA and unmethylated DNA following binding in the endolysosomal environment [47]. In the cytosolic environment, pathogen-derived RNA and DNA may be sensed by receptors such as retinoic acid-inducible gene I (RIG-I) and cyclic GMP-AMP synthase (cGAS) [48,49]. Many viruses maintain a capsid structure not only during intercellular transmission, but also during the early intracellular stages of infection. For instance, the HIV-1 capsid is critical to maintaining post-entry immune quiescence by recruiting host factors and physically shielding the genome from host sensors [50]. In macrophages, failure of the HIV capsid to protect the virus from sensing activates an inflammatory response that prevents viral replication. Adenovirus maintains capsid integrity in the cytosol and uncoats in a sequential and coordinated manner resulting in the viral genome being transferred through the nuclear pore [51]. Infection of primary lung fibroblasts with adenovirus (in the absence of antibody) at a multiplicity of 75 fails to elicit an inflammatory response [6]. This suggests that, like HIV, adenovirus particles are highly effective at shielding viral genomes from DNA sensors. However, TRIM21 neutralization provokes the catastrophic destruction of virus particles that prevents the coordinated uncoating events that must occur for productive infection. Cytoplasmic nucleic acid sensors are able to detect the genomes that are released following TRIM21 neutralization, stimulating a wave of cytokine transcription at around 8 h post-infection that is distinct from initial detection by TRIM21, which peaks at around 4 h post-infection [31]. TRIM21-mediated genome exposure and subsequent sensing has been shown to operate for adenovirus and human rhinovirus-14 (DNA and RNA viruses respectively), with dependencies on cGAS and RIG-I [31]. Thus, the destruction of virus particles in the cytoplasm by TRIM21 potentiates the detection of nucleic acids by innate immune sensors.

## 9. TRIM21 Can Target Diverse Pathogens

The full range of pathogens that are substrates for TRIM21 activity has yet to be determined, but the list is steadily growing. TRIM21 has been shown to exert an inhibitory effect on the survival of intracellular *Salmonella* pre-treated with anti-lipopolysaccharide [52], suggesting that its activity is not limited to viruses. Within viruses, TRIM21 has activity outside of the well-studied adenovirus model. TRIM21 neutralization activity has recently been demonstrated against two picornaviruses: foot-and-mouth disease virus (FMDV) and human rhinovirus-14 (HRV-14) [31,53]. HRV-14 belongs to the major group of rhinoviruses, which enter the cytoplasm via lysis of the endosome [54]. This is in contrast to minor group rhinoviruses which, like many picornaviruses, secrete their RNA genomes to the cytosol through pores in the endosomal membrane [55]. These alternate entry strategies are predicted to influence the accessibility of surface-bound antibodies by TRIM21: translocation of genomic RNA through an endosomal pore is predicted to prevent antibodies from accessing the cytosol. In support of this, TRIM21-dependent innate immune activation was detected for HRV-14 but not for the minor group HRV-2 [31]. The events surrounding FMDV entry are poorly characterised but involve acid-dependent escape from clathrin-coated vesicles [56,57]. This entry strategy potentially releases the antibody-bound viral components into the cytosol, a hypothesis that is consistent with observations of TRIM21-dependent neutralization. However, the inhibitory effect of TRIM21 on picornaviruses owes, at least in part, to its signalling activities: for HRV-14, the block to replication afforded by TRIM21/antibody can be reduced by inhibitors of the NF-κB signalling pathway [31]. Observations of TRIM21-dependent blocks to multi-round viral replication cannot therefore be taken as direct evidence of viral destruction by TRIM21, as the leak of antibodies to the cytosol and subsequent TRIM21-dependent induction of an antiviral state may also contribute.

Experiments using the enveloped virus, respiratory syncitial virus, suggest that detection by TRIM21 is confined to non-enveloped viruses [6]. Mechanistically, this is likely due to the sealing of membranes that follows fusion between viral and cellular membranes, maintaining physical separation of antibodies from the cytoplasm. Viral envelopes therefore represent a defence strategy against transferring host molecules from the outside to the inside of the cell during infection. It is now clear that TRIM21 is able to target diverse pathogens that enter the cell with antibodies attached to their surface. However, the anti-microbial activity of TRIM21 can be thwarted by entry strategies that prevent access of antibodies to the cytosol such as uncoating within the endosome (minor group rhinoviruses) or membrane fusion (enveloped viruses). It remains to be determined whether direct microbial countermeasures may also prevent TRIM21’s activities.

## 10. Intracellular Antibodies in Auto-Immunity

Systemic lupus erythematosus (SLE) is an auto-immune disease characterised by defective clearance of apoptotic bodies and the production of auto-antibodies against a spectrum of targets. Macrophages derived from two SLE mouse models were recently shown to exhibit defects in lysosomal maturation that prolonged residency of immune complexes and other apoptotic debris in the phagolysosomal system [58]. This promoted leakage of membranes, entry of debris and immune complexes to the cytoplasm and activation of immune signalling by cytoplasmic sensors including TRIM21. These findings demonstrate that aberrant entry of antibody to the cytosol can lead to an inappropriate inflammatory danger response via TRIM21, which may contribute to disease pathogenesis. Evidence from other studies suggests that engagement of TRIM21 may represent a common mechanism by which auto-antibodies evoke a pathological response. Auto-antibodies against amphiphysin, a cytosolic membrane binding protein involved in recruitment of dynamin to vesicles at pre-synaptic termini, are associated with the rare, progressive disorder stiff person syndrome (SPS) [59,60]. Passive transfer of anti-amphiphysin from SPS patients can recapitulate pathology in rodents [61], providing compelling support for an etiological role of these auto-antibodies. Anti-amphiphysin antibodies have been observed in the intracellular domain [61,62], although the mechanism by which these antibodies enter the cell is unknown. Nonetheless, intracellular anti-amphyphysin caused aberrant vesicular dynamics in neurons that phenocopied neurons derived from amphiphysin knockout mice. Mechanistically, TRIM21 may elicit the intracellular degradation of amphiphysin, or promote inappropriate immune activation in neurons, thus contributing to the severe clinical outcomes for these patients. Numerous intracellular targets for auto-antibodies have been identified in other patient cohorts. Though the production of auto-antibodies in these cases is believed to be relevant to disease pathogenesis, compelling evidence for their etiologic role is often lacking. Intracellular auto-antigens include glutamic acid decarboxylase GAD65/67 (diabetes mellitus and various CNS disorders including SPS [63,64]), double-stranded DNA (SLE), intraneuronal antigens CDR2, SOX1 and PNMA1 (often arising during paraneoplastic neurological disorders, reviewed in [65]) and TRIM21 itself (Sjögren’s syndrome and SLE; usually referred to as anti-SSA or anti-Ro52). In this latter case, direct contact between TRIM21 and its auto-antibodies could promote the formation of cross-linked immune complexes due to bipolar bridging [4]. However, in all such cases it is unclear whether disease-relevant interactions occur exclusively in the extracellular environment or whether leakage of antibodies into the intracellular environment, and resulting activation of TRIM21, also occurs. Potentially supporting a role for TRIM21 in its physiological, intracellular location is the observation that, as with other Fc receptors [66], polymorphisms in and around the *TRIM21* locus influence susceptibility to SLE [67,68]. A deeper understanding of the location of the pathologically relevant interaction between antibody and auto-antigen, and any role of TRIM21, may be critical to understanding the underlying mechanisms of these diseases.

## 11. Conclusions

TRIM21 patrols an environment that is devoid of antibodies under normal physiological circumstances. Its role in defending against infection by non-enveloped viruses is now well established in in vitro neutralization assays and in mouse models of infection. However, it is possible that antibodies reach the cytosol in other pathological circumstances, including during auto-immunity. By virtue of its broad tissue expression and ultra-high affinity for Fc, it is likely that TRIM21 will engage cytosolic antibodies whenever they occur. Intracellular antibodies may therefore have important roles both in defending the cytosol against infection and during pathological responses to intracellular auto-antigens.
